# The Charlson Comorbidity Index is associated with risk of 30-day mortality in patients with myocardial injury after non-cardiac surgery

**DOI:** 10.1038/s41598-021-98026-4

**Published:** 2021-09-23

**Authors:** Sojin Kim, Jungchan Park, Ji-Hye Kwon, Ah Ran Oh, Joonhee Gook, Kwangmo Yang, Jin-ho Choi, Kyunga Kim, Ji Dong Sung, Joonghyun Ahn, Seung-Hwa Lee

**Affiliations:** 1 Department of Anesthesiology and Pain Medicine, Samsung Medical Center, Sungkyunkwan University School of Medicine, Seoul, Korea; 2 Department of Biomedical Sciences, Ajou University Graduate School of Medicine, Suwon, Korea; 3 Department of Anesthesiology and Pain Medicine, Kangwon National University Hospital, Chuncheon, Korea; 4 Center for Health Promotion, Samsung Medical Center, Sungkyunkwan University School of Medicine, Seoul, Korea; 5 Department of Emergency Medicine, Samsung Medical Center, Sungkyunkwan University School of Medicine, Seoul, Republic of Korea; 6 Statistics and Data Center, Research Institute for Future Medicine, Samsung Medical Center, Seoul, Korea; 7 Department of Digital Health, SAIHST, Sungkyunkwan University, Seoul, Korea; 8 Rehabilitation and Prevention Center, Heart Vascular Stroke Institute, Samsung Medical Center, Sungkyunkwan University School of Medicine, 81 Irwon-ro, Gangnam-gu, Seoul, Korea; 9 Department of Biomedical Engineering, Seoul National University College of Medicine, Seoul, Korea

**Keywords:** Cardiovascular diseases, Myocardial infarction

## Abstract

Myocardial injury after non-cardiac surgery (MINS) is a well-known and relevant indicator of early postoperative mortality, but factors related to increased mortality in MINS patients are as yet unknown. The Charlson Comorbidity Index (CCI) is widely used to classify various comorbid conditions and underlying diseases. Our study aimed to determine the prognostic value of CCI with regard to mortality of patients with MINS. This study comprises 5633 patients who had MINS as diagnosed by a rise of postoperative cardiac troponin I above the normal range (≥ 0.04 ng/mL) from January 2010 to June 2019. Patients were divided into two groups according to median weighted CCI score: low CCI (≤ 2) and high CCI (> 2) groups. The primary outcome was 30-day mortality after surgery, and secondary outcomes were 1-year and overall mortalities. Of the 5633 patients, 3428 (60.9%) were in the low CCI group (1.21 ± 0.84) and 2205 (39.1%) were in the high CCI group (4.17 ± 1.82). After propensity score matching, mortality during the first 30 days after surgery was significantly greater in the high CCI group than the low CCI group (9.4% vs. 6.0%, respectively; hazard ratio 1.56, 95% confidence interval 1.23–1.98, *p* < 0.001). A high CCI score was associated with increased 30-day mortality in patients with MINS, suggesting that the CCI may need to be considered when predicting outcomes of MINS patients.

## Introduction

The presence of comorbid conditions is associated with reduced survival of patients in various clinical situations^1–4^. The Charlson Comorbidity Index (CCI) was introduced in 1987 to classify the severity of comorbid conditions^5^. Since then, it has been validated as a reliable tool for predicting prognosis and has been updated with different weights for various diseases^6,7^. Recent CCI scoring systems with updated weights have been reported to correlate well with the prognoses of critically ill patients and those undergoing surgical procedures^8^. The CCI is one of the most widely used stratification tools familiar to clinicians in different medical fields and is credited highly for its validation in an extensive database, especially when mortality is the outcome of interest^9^.

Myocardial injury after non-cardiac surgery (MINS) is associated with adverse postoperative outcomes including death, non-fatal cardiac arrest, dysfunction, congestive heart failure, and stroke^10^. MINS is diagnosed in the presence of postoperative cardiac troponin (cTn) elevation related to ischemia within 30 days after non-cardiac surgery, with or without ischemic symptoms^11^. The incidence of MINS for all non-cardiac surgeries has been estimated to be as high as 17.9%, and comorbidities such as hypertension, coronary artery disease, heart failure, prior myocardial infarction, and kidney disease significantly increase the risk of MINS^12^. However, it is unclear whether the effects of comorbidities extend to the short-term mortality of patients after MINS diagnosis. Therefore, in this study, we used the CCI to grade the severity of comorbidities in MINS patients and evaluated the incidence of postoperative 30-day mortality.

## Results

### Patient characteristics

A total of 43,019 patients undergoing non-cardiac surgery between January 2010 and June 2019, were identified in the registry. Among these patients, we excluded (1) 1154 patients younger than 18 years at the time of surgery (2) 35,990 patients with no postoperative cTn measurement or elevation and (3) 242 patients with cTn elevation due to non-ischemic etiology.

**Figure 1:**
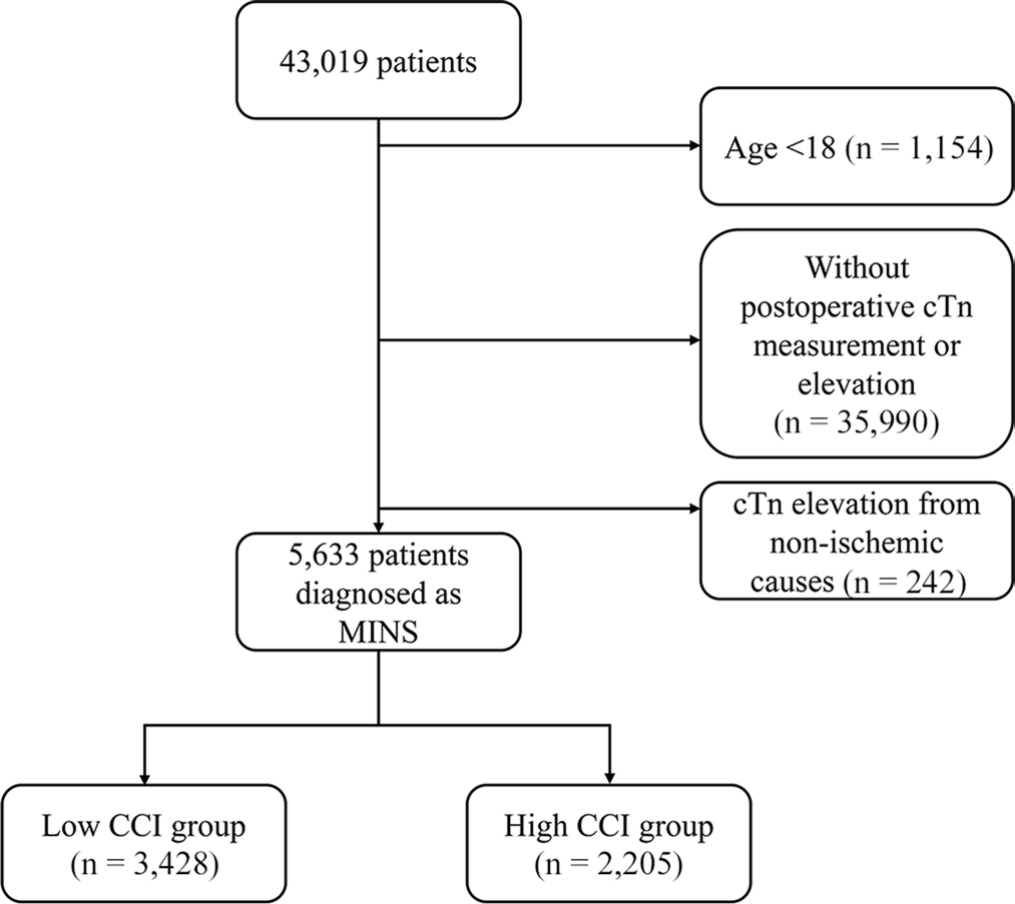
Flowchart of study patients.

The final study cohort consisted of 5633 patients diagnosed with MINS. These patients were then stratified into two groups according to median weighted CCI value: 3428 (60.9%) patients were allocated to the low CCI group and 2205 (39.1%) patients to the high CCI group. A flowchart of the present study is presented in Fig. 1. Propensity score matching produced 1857 well-balanced pairs with an ASD value lower than 10%. Baseline characteristics of the patients of the entire and propensity score matched populations are listed in Table 1. The mean CCI value was 1.21 ± 0.8 in the low group and 4.17 ± 1.8 in the high group. The breakdown of CCI values for each group is described in Supplementary Table S1. The high CCI group had a larger proportion of male, hypertension, arrhythmia, preoperative use of continuous renal replacement therapy, beta-blocker and direct oral anticoagulant use, high-risk surgeries according to ESC/ESA criteria, general anesthesia, longer operation duration, and continuous infusion of an inotropic agent or a red blood cell transfusion during surgery. The low CCI group had more patients with a history of smoking and emergency operation. The most common types of surgery were orthopedic (20.2%), gastrointestinal (16.7%), and non-cardiac thoracic (16.3%) in the low CCI group and gastrointestinal (35.2%), transplantation (15.2%), and non-cardiac thoracic (14.9%) surgery in the high CCI group (Supplementary Table S2).

**Table 1:** Baseline characteristics of entire and propensity score matched population.

	Entire population				Propensity score matched population			
	Low CCI group (n = 3428)	High CCI group (n = 2205)	*p* value	ASD (%)	Low CCI group (n = 1857)	High CCI group (n = 1857)	*p* value	ASD (%)
CCI (mean (SD))*	1.21 (± 0.8)	4.17 (± 1.8)	< 0.001	> 99	1.47 (± 0.77)	4.18 (± 1.83)		
Peak cTn I level*	3.16 (± 23.7)	2.15 (± 14.9)	0.073	5.1	1.98 (± 12.9)	2.14 (± 15.4)		
Age	65.3 (± 14.6)	65.3 (± 12.7)	0.976	0.1	65.7 (± 13.4)	65.5 (± 12.8)	0.604	1.0
Male sex	1936 (56.5)	1432 (64.9)	< 0.001	17.4	1151 (62.0)	1183 (63.7)	0.292	3.6
Current smoking	330 (9.6)	176 (8.0)	0.039	5.8	144 (7.8)	161 (8.7)	0.339	3.3
Current alcohol	545 (15.9)	272 (12.3)	< 0.001	10.2	237 (12.8)	237 (12.8)	> 0.999	< 0.1
Cardiac morbidity								
Hypertension	2226 (64.9)	1513 (68.6)	0.005	7.8	1218 (65.6)	1240 (66.8)	0.466	2.5
Coronary artery disease	808 (23.6)	503 (22.8)	0.532	1.8	441 (23.7)	437 (23.5)	0.908	0.5
Arrythmia	311 (9.1)	258 (11.7)	0.002	8.6	194 (10.4)	200 (10.8)	0.790	1.0
Valvular heart disease	74 (2.2)	41 (1.9)	0.497	2.1	37 (2.0)	34 (1.8)	0.811	1.2
Preoperative care								
Intensive care unit	351 (10.2)	258 (11.7)	0.093	4.7	198 (10.7)	207 (11.1)	0.674	1.6
ECMO	1 (0.0)	0 (0.0)	> 0.999	2.4	0 (0.0)	0 (0.0)		< 0.1
CRRT	24 (0.7)	38 (1.7)	0.001	9.4	22 (1.2)	26 (1.4)	0.663	1.9
Ventilator	84 (2.5)	52 (2.4)	0.869	0.6	42 (2.3)	42 (2.3)	> 0.999	< 0.1
Operative variables								
ESC/ESA surgical high risk	658 (19.2)	767 (34.8)	< 0.001	35.7	470 (25.3)	536 (28.9)	0.016	8.0
Emergency operation	1034 (30.2)	549 (24.9)	< 0.001	11.8	484 (26.1)	479 (25.8)	0.881	0.6
General anesthesia	2842 (82.9)	2022 (91.7)	< 0.001	26.7	1647 (88.7)	1677 (90.3)	0.121	5.3
Operation duration, hours	3.07 (± 2.49)	4.11 (± 2.99)	< 0.001	37.8	3.55 (± 2.67)	3.81 (± 2.92)	0.004	9.5
Continuous infusion of inotropics	1305 (38.1)	1013 (45.9)	< 0.001	16.0	758 (40.8)	804 (43.3)	0.135	5.0
RBC transfusion	325 (9.5)	520 (23.6)	< 0.001	38.7	289 (15.6)	358 (19.3)	0.003	9.8
Preoperative use of								
Beta-blocker	946 (27.6)	731 (33.2)	< 0.001	12.1	568 (30.6)	583 (31.4)	0.619	1.7
Calcium channel blocker	1211 (35.3)	816 (37.0)	0.210	3.5	699 (37.6)	688 (37.0)	0.734	1.2
RAAS inhibitor	1355 (39.5)	915 (41.5)	0.149	4.0	760 (40.9)	774 (41.7)	0.665	1.5
Statin	1177 (34.3)	755 (34.2)	0.965	0.2	629 (33.9)	638 (34.4)	0.782	1.0
Antiplatelet agent	1312 (38.3)	884 (40.1)	0.181	3.7	726 (39.1)	740 (39.8)	0.663	1.5
Direct oral anticoagulant	51 (1.5)	53 (2.4)	0.017	6.6	31 (1.7)	43 (2.3)	0.196	4.6
Warfarin	220 (6.4)	157 (7.1)	0.330	2.8	125 (6.7)	138 (7.4)	0.443	2.7

Data are presented as *n* (%) or mean (± standard deviation).

*CCI* Charlson Comorbidity Index, *ASD* Absolute standardized mean difference, *ECMO* Extracorporeal membranous oxygenation, *CRRT* Continuous renal replacement therapy, *ESC* European Society of cardiology, *ESA* European Society of Anaesthesiology, *RBC* Red blood cell, *RAAS* Renin–angiotensin–aldosterone system.

*These two variables were not retained in the propensity score matching.

### Clinical outcomes

Mortality during the first 30 postoperative days in the entire study population was 7.5% (415/5633), and the high CCI group had a significantly higher risk of 30-day mortality compared to the low CCI group before and after multivariable adjustment (9.5% vs. 6.0%, hazard ratio (HR) 1.58, 95% confidence interval (CI) 1.30–1.92, *p* < 0.001 for univariable analysis and HR 1.72, 95% CI 1.39–2.13, *p* < 0.001 for multivariable analysis) (Table 2). In the propensity matched group, mortality in the 30-day follow-up was higher in the high CCI group than in the low CCI group (9.4% vs. 6.0%, HR 1.56, 95% CI 1.23–1.98, *p* < 0.001). Mortalities during the one-year and overall follow-up periods were higher in the high CCI group than the low CCI group (25.9% vs. 17.0%, HR 1.57, 95% CI 1.36–1.81, *p* < 0.001 for one-year mortality and 40.5% vs. 26.9%, HR 1.61, 95% CI 1.44–1.80, *p* < 0.001 for overall mortality) (Table 2). Of note, the median duration for overall mortality was 2.54 years. Postoperative diagnosis and management of patients experiencing MINS are listed in Supplementary Table S3. Postoperative treatments were mostly based on ischemic symptoms or signs of infarction. In further analyses, MINS patients were stratified according to quartiles of CCI. Mean CCI values were 0.47 ± 0.50, 2.00 ± 0, 3.00 ± 0, and 5.60 ± 1.91 for the 1st, 2nd, 3rd, and 4th quartile, respectively. Baseline characteristics of groups according to quartiles are shown in Supplementary Table S4. Compared with the 1st quartile, 30-day mortality was higher in the 3rd and 4th quartile (9.5% vs. 5.4%; HR 1.76, 95% CI 1.35–2.32, *p* < 0.001 for the 3rd quartile and 9.4% vs. 5.4%, HR 1.75, 95% CI 1.32–2.33, *p* < 0.001 for the 4th quartile) (Table 3). To examine the influence of the CCI on mortality, additional analysis was performed in patients that are not detected as MINS. In a univariable analysis of the patients that are not detected as MINS, the CCI was not related to 30-day mortality (1.3% in the low CCI group vs. 1.2% in the high CCI group, unadjusted HR 0.93, 95% CI 0.76–1.14, *p* = 0.48) but was related to mortality during one year or the overall follow-up period (Supplementary Table S5).

**Table 2:** Clinical outcomes of entire and propensity score matched population.

	Low CCI group	High CCI group	Unadjusted HR (95% CI)	*p* value	*Adjusted HR (95% CI)	*p* value
**Entire population**	**(n = 3428)**	**(n = 2205)**				
30-day mortality	206 (6.0)	209 (9.5)	1.58 (1.30–1.92)	< 0.001	1.72 (1.39–2.13)	< 0.001
One-year mortality	505 (14.7)	574 (26.0)	1.80 (1.60–2.03)	< 0.001	1.78 (1.56–2.03)	< 0.001
Overall mortality	763 (22.3)	905 (41.0)	1.94 (1.76–2.13)	< 0.001	1.83 (1.64–2.03)	< 0.001
**Propensity score matched population**	**(n = 1857)**	**(n = 1857)**				
30-day mortality	112 (6.0)	174 (9.4)			1.56 (1.23–1.98)	< 0.001
One-year mortality	316 (17.0)	481 (25.9)			1.57 (1.36–1.81)	< 0.001
Overall mortality	500 (26.9)	753 (40.5)			1.61 (1.44–1.80)	< 0.001

*Covariates included age, sex, hypertension, alcohol, general anesthesia, emergency operation, use of RAAS inhibitor, statin, active cancer, and preoperative intensive care unit management.

**Table 3:** Clinical outcomes according to quartiles of the CCI.

	1st quartile (n = 1766)	1st vs 2nd quartile (n = 1662)	1st vs 3rd quartile (n = 1210)	1st vs 4th quartile (n = 995)
30-day mortality, No (%)	95 (5.4)	111 (6.7)	115 (9.5)	94 (9.4)
Unadjusted HR (95% CI)	1 [reference]	1.23 (0.94–1.50)	1.76 (1.35–2.32)	1.75 (1.32–2.33)
* p* value		0.13	< 0.001	< 0.001
One-Year mortality, No (%)	156 (8.8)	349 (21.0)	324 (26.8)	250 (25.1)
Unadjusted HR (95% CI)	1 [reference]	2.37 (1.96–2.86)	3.07 (2.54–3.72)	2.89 (2.37–3.53)
* p* value		< 0.001	< 0.001	< 0.001
Overall mortality, No (%)	216 (12.2)	547 (32.9)	515 (42.6)	390 (39.2)
Unadjusted HR (95% CI)	1 [reference]	2.74 (2.34–3.21)	3.66 (3.12–4.29)	3.40 (2.88–4.02)
* p* value		< 0.001	< 0.001	< 0.001

Values are n (%).

*CCI* Charlson Comorbidity Index, *HR* hazard ratio, *CI* confident interval.

**Figure 2:**
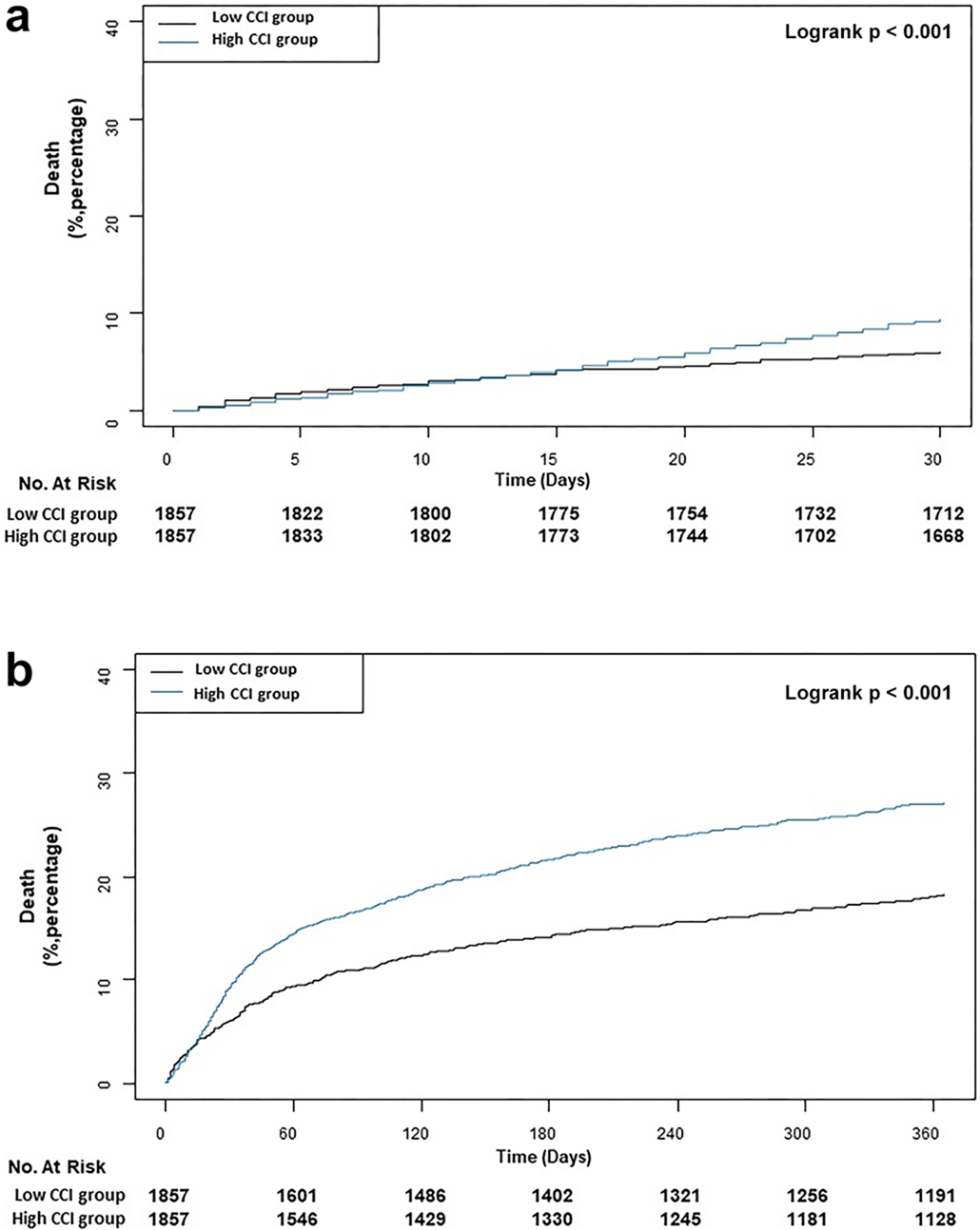
Kaplan–Meier curves of (**a**) 30-day mortality and (**b**) One-year mortality for the propensity score matched population.

**Figure 3:**
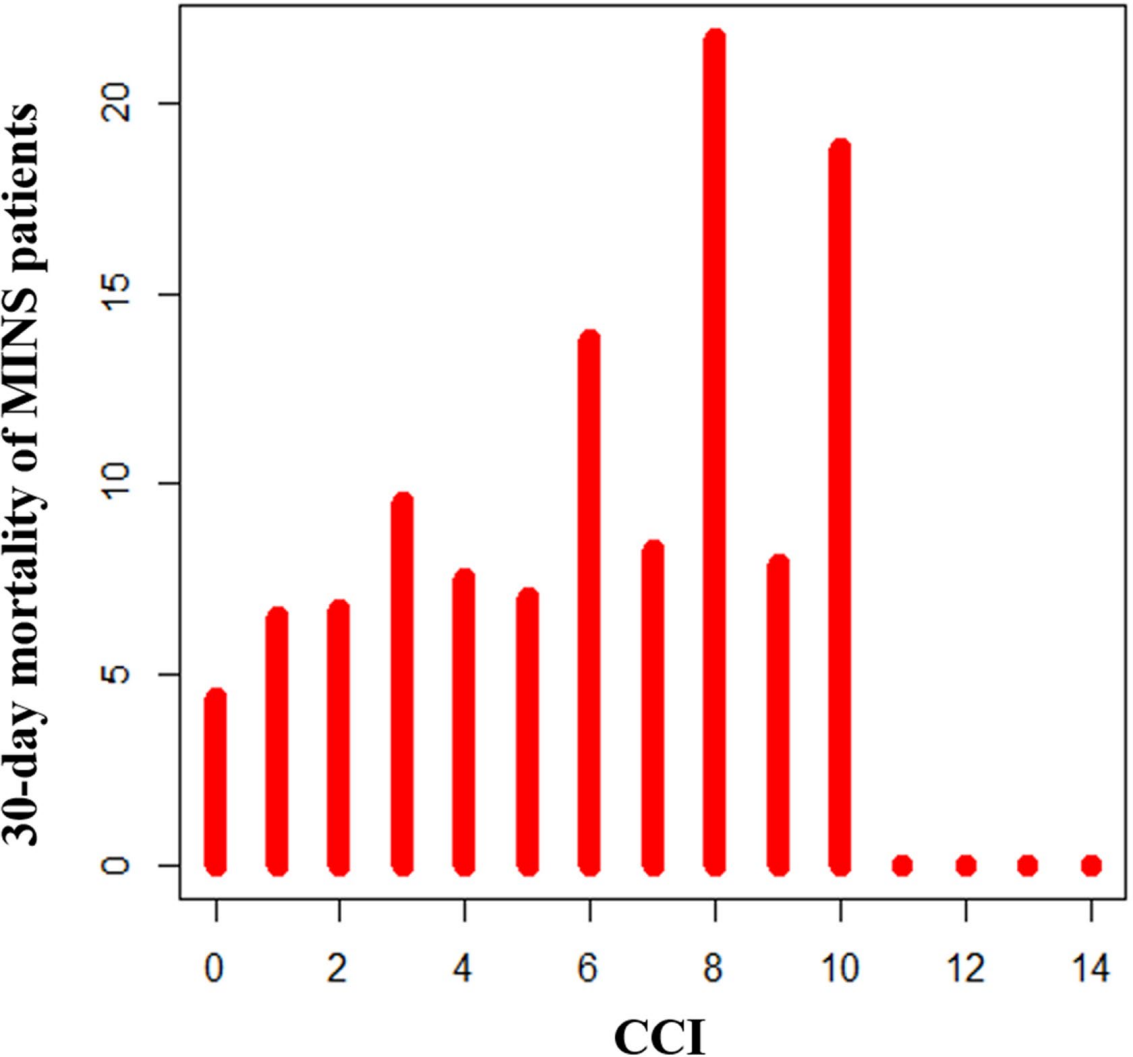
Histogram showing the association between CCI as a continuous variable and 30-day mortality of MINS.

**Figure 4:**
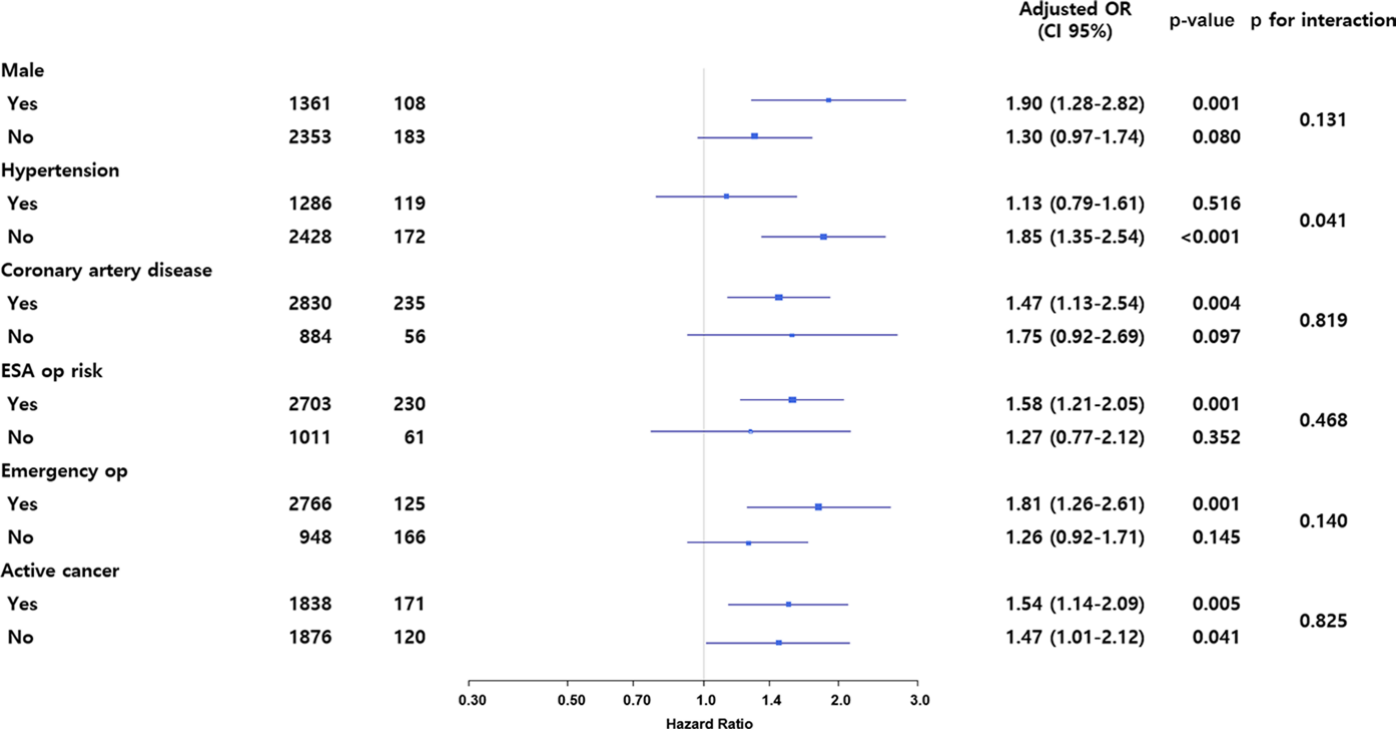
A forest plot based on subgroup analysis.

**Figure 5:**
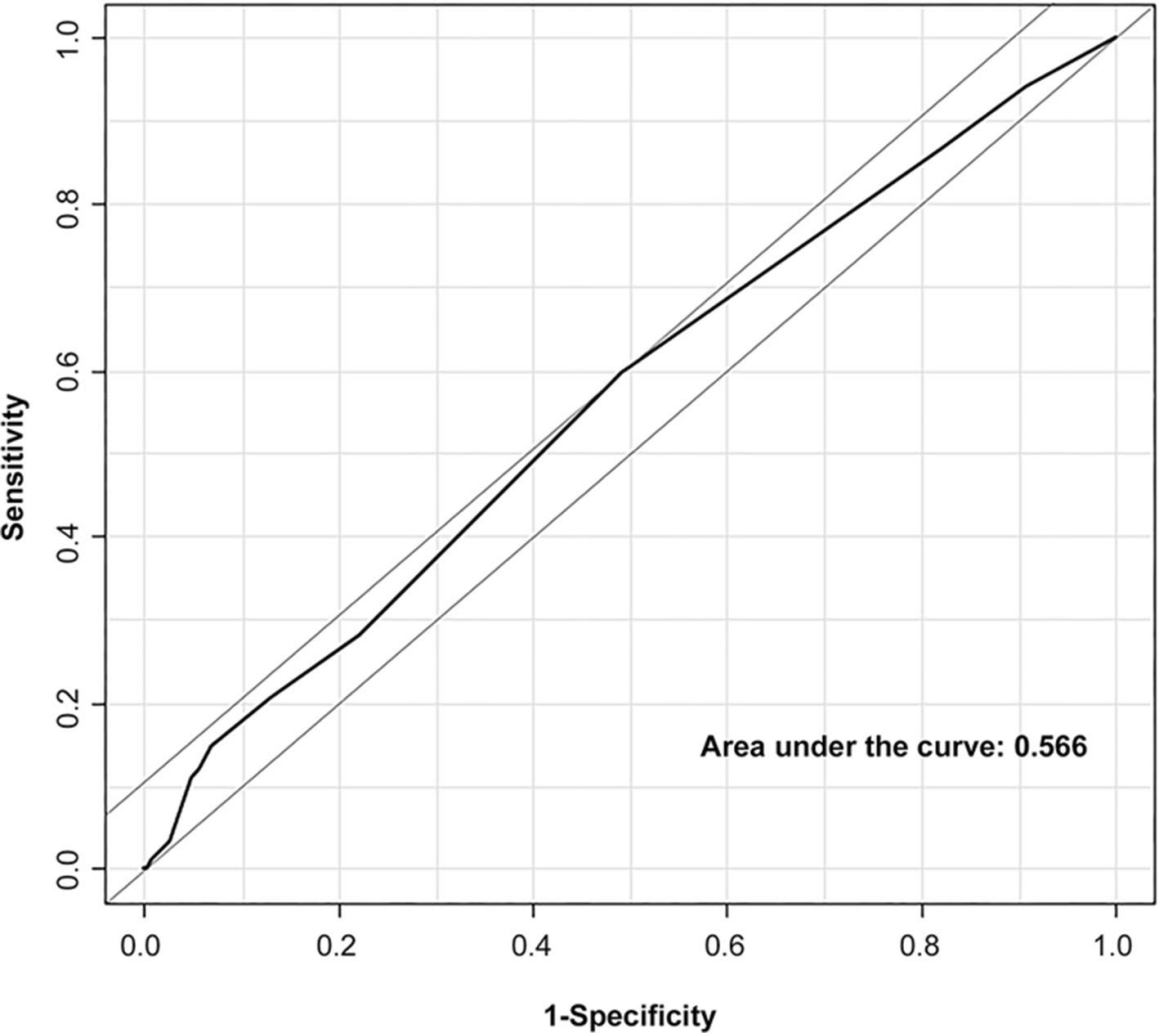
Receiver-operating characteristic plots to estimate the threshold of the Charlson Comorbidity Index associated with 30-day mortality.

Kaplan–Meier curves for survival suggested that patients in the high CCI group had significantly higher mortalities during the 30-day and one-year follow-ups (Fig. 2a,b). A histogram showing the association between the CCI as a continuous variable and 30-day mortality of MINS is shown in Fig. 3. Subgroup analysis demonstrated that the association between CCI and MINS mortality was significantly affected by hypertension (*p* for interaction = 0.04), and 30-day mortality of MINS was significantly increased by high CCI only in patients without hypertension (HR 1.85, 95% CI 1.35–2.54, *p* < 0.001 for without hypertension and HR 1.13, 95% CI 0.79–1.61, *p* = 0.52 for with hypertension) (Fig. 4). Sensitivity analysis showed that this association was significant under all circumstances (Supplementary Table S6). In the receiver-operating characteristic (ROC) plot, the threshold of CCI association with 30-day mortality was 2, with an area under the ROC curve of 0.566 (Fig. 5). The sensitivity and specificity of the estimated threshold were 59.8% and 50.8%, respectively.

## Discussion

In this observational study, MINS patients with a higher CCI score showed a significant increase in mortality during the first 30 days after surgery compared to those with a lower CCI score. In addition, one-year mortality and overall mortality were also significantly higher in the high CCI group. Our finding suggests that CCI may help identify MINS patients who are at higher mortality risk.

The CCI is a valid tool for predicting the survival of patients based on comorbidities. It is simple to calculate and readily applicable to any patient, including those undergoing surgery, and can be easily obtained from International Classification of Diseases (ICD) code or an individual’s electronic medical records^13^. The original version featured 17 conditions and assigned weights for each condition based on the adjusted relative risk of one-year mortality^5^. This comorbidity index has been verified to assess mortality and prognosis^14^. Owing to the advances in medical treatment and disease management, weights were reassigned to 12 variables based on ICD-10 code in the updated version in 2011^9^. Five variables, including myocardial infarction, were reassigned to a weight of 0 (Supplementary Table S7). In this study, the updated version of the CCI was used to evaluate whether this index is associated with mortality of MINS patients. Since the association between comorbidity and long-term outcomes has been verified in general surgical patients, we focused on 30-day mortality in MINS patients as the primary endpoint. In addition, previous studies have shown that comorbidity is related to the development of MINS, but the relationship between comorbidity and mortality after diagnosis of MINS has not been evaluated thoroughly.

MINS is an independent predictor of 30-day mortality and the most common perioperative cardiovascular complication^9^. It is defined as a myocardial injury caused by ischemia, with at least one value of cardiac troponin elevation above the 99th percentile upper reference limit, without the requirement of ischemic symptoms^10,15,16^. Myocardial ischemia can be caused by pre-existing coronary disease or thrombosis or myocardial oxygen supply and demand mismatch^17^. Considering the etiology of MINS, antithrombotic agents (i.e., rivaroxaban) and anticoagulants (i.e., dabigatran) can be used for treatment. Aspirin and statin therapies and early cardiology assessment and intervention of patients decrease 30-day mortality of MINS patients^17,18^.

Although cTn is a sensitive and specific biomarker of cardiac myocyte damage, ischemic injury is not the only cause of cTn elevation. A broad spectrum of diseases, including non-coronary and even non-cardiac conditions, is associated with cTn elevation^19^. Patients with comorbidities are vulnerable to MINS despite diagnostic criteria that exclude cTn elevation with a definite non-ischemic cause^12^. This study delineates the impact of comorbidities on the increased 30-day mortality in MINS patients as not only a consequence of acute coronary ischemic changes, but also accounting for the poor general conditions and metabolic burdens, graded by the CCI scoring system. Our results also indicate that comorbidities stratified by the CCI are associated with mortality after diagnosis of MINS, and that this association is valid even in the short-term follow-up period. Moreover, the conclusion was supported by a conflicting result in patients without MINS, in whom the CCI score was not an independent predictor of 30-day mortality. The detrimental effects of comorbidities included in the CCI were significant only in patients diagnosed with MINS.

We further stratified the patients into four groups according to quartile of CCI. The results showed that, compared with the 1st quartile group, 30-day mortality was significantly higher in the 3rd and 4th quartile groups, with no significant difference between the 1st and 2nd quartile groups. This implies that MINS mortality is affected by comorbidities only when they exceed a certain severity. Therefore, we estimated the threshold associated with 30-day mortality, which was a CCI of 2. However, the area under the curve was relatively low, as were the sensitivity and specificity. In addition, subgroup analysis revealed an association between high CCI and MINS mortality only in patients without a history of hypertension. Hypertension is not one of the 12 components of CCI, and the result of subgroup analysis implies that hypertension can affect the association between mortality due to MINS and other CCI components. This suggests that comorbidities not included in the CCI should also be considered for the incidence of 30-day mortality in MINS patients. Due to the conflicting results from the subgroup analysis and the limitation of the low ROC, the CCI cannot be relied upon solely when assessing mortality in patients with MINS. However, our study shows that patients with higher CCI have a significantly higher risk of mortality than those with lower CCI, emphasizing the need for patient surveillance and intensive treatment for MINS patients with high CCI.

This study had several limitations. First, due to the nature of a retrospective, observational study, the results might have been influenced by undiscovered confounding factors, despite the use of statistical adjustment including propensity matching and multivariable analysis. Second, the analysis included only patients with available cTn measurements, which were performed selectively. Our results from selected patients with known risk might not be generalizable to all patients. Last, detailed preoperative cardiac evaluations other than cTn level, such as left ventricular ejection fraction or coronary artery angiograms, were unavailable for all patients. Our results may not be sufficient to recommend a routine adaptation of CCI to fully predict mortality of MINS patients, but still suggest that upon using the CCI, the severity of the individual’s comorbidity and associated risks can be estimated. Higher CCI may encourage clinicians to intervene earlier than lower CCI, in order to avoid further adverse events including death.

## Materials and methods

This study is a retrospective chart review and was approved by the Institutional Review Board of Samsung Medical Center (SMC 2019-08-048). The need for written informed consent for access to the registry was waived by the Institutional Review Board of Samsung Medical Center as the entire dataset was extracted in de-identified form. The present study was conducted in reference to the precepts of the Declaration of Helsinki, and results are reported according to “Strengthening the Reporting of Observational Studies in Epidemiology”^20^.

All pre-, intra-, and postoperative data were obtained from the SMC-TINCO registry (Samsung Medical Center-Troponin in Non-cardiac Operations, KCT0004244), which contains the data of 43,019 consecutive patients with at least one cTn I measurement during a preoperative evaluation or within 30 postoperative days of non-cardiac surgery between January 2010 and June 2019, at Samsung Medical Center, Seoul, South Korea. Data were generated using “Clinical Data Warehouse Darwin-C,” an institutional electronic medical archive system for retrieval of de-identified medical and administrative records. Mortality statistics were derived from our institution’s medical records; mortalities in other institutions were verified with the National Population Registry of the Korea National Statistical Office.

### Study population, definitions, and endpoints

For this study, we identified patients who were diagnosed with MINS and allocated them into two groups according to median CCI value: patients with CCI ≤ 2 (low group) and those with CCI > 2 (high group). MINS was defined as an elevation of cTn I level as measured by an automated analyzer (Advia Centaur XP, Siemens Healthcare Diagnostics, Erlangen, Germany) above the 99th percentile of the upper reference limit within 30 postoperative days. The lowest measurable value was 6 ng/L, and the 99th percentile cutoff for cTn I was 40 ng/L^21^. Following current diagnostic criteria, cTn elevation with evidence of a non-ischemic etiology such as pulmonary embolism, atrial fibrillation, cardioversion, sepsis, or chronic cTn elevation was not considered MINS^15^. The comorbidity of each patient was quantified using Quan et al.’s updated version of CCI, which is comprised of a summation score of 12 medical conditions with varying weights assigned based on chart review or derived from administrative data with ICD-10 codes^8^. A score of 1 was given for chronic pulmonary disease, diabetes with chronic complications, rheumatologic disease, and renal disease; a score of 2 was assigned to congestive heart failure, dementia, mild liver disease, hemi or paraplegia, any malignancy including leukemia and lymphoma; a score of 4 was used for moderate to severe liver disease and acquired immunodeficiency syndrome; and a score of 6 was assigned to a metastatic solid tumor^8^. The sum of all comorbidities yields a single comorbidity score for a patient, with a value of 0 indicating no comorbidity and higher values indicating more severe comorbidity^8^. High-risk surgery was classified as that with a reported risk of mortality greater than 5% according to the 2014 European Society of Cardiology/European Society of Anesthesiology (ESC/ESA) guidelines^22^.

The primary outcome was all-cause mortality during 30 postoperative days, and the mortalities during one year and overall follow-up were compared as secondary outcomes.

### Perioperative management and cTn I measurement

Perioperative management including anesthetic practice and perioperative care follow institutional protocols, which are based on current guidelines. In our hospital, perioperative cTn I measurements were not performed routinely in non-cardiac surgery but were performed selectively at the recommendation of the cardiologist or for patients with one or more major cardiovascular risk factors, such as a history of heart failure, ischemic heart disease, stroke, transient ischemic attack, diabetes mellitus on insulin therapy, or chs3 ronic kidney disease, or for those undergoing high-risk surgery. When cTn elevation was detected, patients were referred to cardiologists for further workup (e.g., electrocardiograms, coronary angiography) or received secondary preventive drugs.

### Statistical analysis

Baseline characteristics are presented as means ± standard deviations (SD) or medians with interquartile ranges (IQRs) for continuous variables and as numbers (%) for categorical variables. For continuous data, differences were compared by t-test or Mann–Whitney test, and categorical data were compared using the chi-square or Fisher’s exact test. Mortalities were compared using Cox regression analysis; results are reported as HRs and 95% confidence intervals CIs. Clinically relevant variables and those with a *p*-value < 0.05 were retained in the multivariable Cox regression model, comprising sex, operation duration, alcohol consumption, high-risk operation, general anesthesia, emergency surgery, preoperative use of a beta-blocker, intraoperative use of continuous inotropics, and red blood cell transfusion. To reduce selection bias and the effects of potential confounding factors, a 1:1 propensity score matched analysis without replacement was conducted. Absolute standardized mean differences (ASD) lower than 10% were regarded as well-balanced variables between groups. Kaplan–Meier estimates were conducted for survival analysis, and these were compared with the log-rank test. Subgroup analysis was conducted to reveal any interactions of male sex, coronary artery disease, hypertension, emergency operation, high-risk operation, and active cancer with relevant factors and is presented as a forest plot. To evaluate the capability of CCI to predict 30-day mortality, Pearson’s correlation coefficient was estimated. A ROC plot was constructed to calculate the threshold CCI score and to compute its specificity and sensitivity as a predictive factor. The potential impact of unmeasured confounders was estimated in the sensitivity analysis. This analysis estimates the effect of an unmeasured binary confounder on the measured causal association between a binary exposure and a binary outcome assuming that the prevalence of the unmeasured confounder is 40%^23^. The power of this study for the given sample size was evaluated using Spearman's rank correlation^24^ and was 0.91 and 0.98 for estimated HRs of 1.4 and 1.5, respectively. All statistical analyses were performed with R 4.0.2 (Vienna, Austria; http://www.R-project.org/) using the PowerSurvEpi package. All reported *p*-value were two-tailed, and statistical significance was assumed at *p*-value < 0.05.

## Supplementary Information

Supplementary Information.
