# Supplementary material for: The Charlson Comorbidity Index is associated with risk of 30-day mortality in patients with myocardial injury after non-cardiac surgery
Source: Sci Rep. 2021 Sep 23;11:18933. doi: 10.1038/s41598-021-98026-4 (PMC8460655; doi:10.1038/s41598-021-98026-4)
Supplement: Supplementary file 1 — Supplementary Information. [file 41598_2021_98026_MOESM1_ESM.pdf]

The Charlson Comorbidity Index is associated with risk of 30-day mortality in patients with myocardial injury after non-cardiac surgery

Short title: CCI and mortality of MINS

Sojin Kim, MD[1], Jungchan Park, MD[1], Ji-Hye Kwon, MD[1], Ah Ran Oh, MD[1], Joonhee Gook, MD[1], Kwangmo Yang, MD[2], Jin-ho Choi, MD, PhD[3], Kyunga Kim, PhD[4,5], Ji Dong Sung, MD, PhD[6], Joonghyun Ahn[4] and Seung-Hwa Lee*, MD,[6,7]

[1]Department of Anesthesiology and Pain Medicine, Samsung Medical Center, Sungkyunkwan University School of Medicine, Seoul, Korea

[2]Center for Health Promotion, Samsung Medical Center, Sungkyunkwan University School of Medicine, Seoul, Korea

[3]Department of Emergency Medicine, Samsung Medical Center, Sungkyunkwan University School of Medicine, Seoul, Republic of Korea

[4]Statistics and Data Center, Research Institute for Future Medicine, Samsung Medical Center, Seoul, Korea

[5]Department of Digital Health, SAIHST, Sungkyunkwan University, Seoul, Korea

[6]Rehabilitation & Prevention Center, Heart Vascular Stroke Institute, Samsung Medical Center, Sungkyunkwan University School of Medicine, Seoul, Korea

[7]Department of Biomedical Engineering, Seoul National University College of Medicine

Supplementary Table S1. Baseline characteristics of patients according to the CCI

	**Low CCI group (*n* = 3428)**	**High CCI group (*n* = 2205)**	***P*-value**
Chronic pulmonary disease	115 (3.4)	231 (10.5)	<0.001
Rheumatologic disease	29 (0.8)	20 (0.9)	0.925
Diabetes with chronic complications	915 (26.7)	1658 (75.2)	<0.001
Renal disease	401 (11.7)	367 (16.6)	<0.001
Congestive heart failure	45 (1.3)	161 (7.3)	<0.001
Dementia	56 (1.6)	157 (7.1)	<0.001
Mild liver disease	59 (1.7)	561 (25.4)	<0.001
Hemiplegia or paraplegia	7 (0.2)	34 (1.5)	<0.001
Any malignancy, including leukemia and lymphoma	1181 (34.5)	1821 (82.6)	<0.001
Moderate or severe liver disease	0 (0.0)	97 (4.4)	<0.001
AIDS/HIV	0 (0.0)	1 (0.0)	0.824
Metastatic solid tumor	0 (0.0)	178 (8.1)	<0.001

CCI: Charlson Comorbidity Index, AIDS: Acquired immune deficiency syndrome, HIV: Human immunodeficiency virus

Supplementary Table S2. Types of surgery

	**Low CCI group (n = 3428)**	**High CCI group (n = 2205)**
Vascular	468 (13.7)	106 (4.8)
Orthopedic	691 (20.2)	150 (6.8)
Neuro	426 (12.4)	261 (11.8)
Breast or Endo	76 (2.2)	41 (1.9)
Plastic or Otolaryngeal or Eye	138 (4.0)	67 (3.0)
Transplantation	178 (5.2)	335 (15.2)
Gynecology or Urology	298 (8.7)	129 (5.9)
Gastrointestinal	574 (16.7)	776 (35.2)
Non-cardiac thoracic	558 (16.3)	329 (14.9)
Others	21 (0.6)	11 (0.5)

CCI: Charlson Comorbidity Index

Supplementary Table S3. Postoperative diagnosis and management in MINS patients

	Low CCI group (n = 3428)	High CCI group (n = 2205)	p-value
Postoperative cardiac consultation	1290 (37.6)	773 (35.1)	0.054
Postoperative evaluation			
Echocardiogram	0 (0.0)	1 (0.0)	0.824
Stress echocardiogram	5 (0.1)	4 (0.2)	>0.999
Treadmill test	11 (0.3)	1 (0.0)	0.058
Coronary computed tomographic angiography	37 (1.1)	13 (0.6)	0.077
Coronary artery angiogram	198 (5.8)	114 (5.2)	0.362
Postoperative diagnosis			
Myocardial infarction	81 (2.4)	40 (1.8)	0.196
ST-elevation	11 (0.3)	8 (0.4)	0.976
Non ST-elevation	70 (2.0)	32 (1.5)	0.128
Postoperative care			
Percutaneous coronary intervention	98 (2.9)	56 (2.5)	0.527
Coronary artery bypass grafting	19 (0.6)	6 (0.3)	0.177
Intensive care unit	1962 (57.2)	1607 (72.9)	<0.001
ECMO	2 (0.1)	0 (0.0)	0.682
Continuous renal replacement therapy	59 (1.7)	67 (3.0)	0.002
Ventilator	788 (23.0)	684 (31.0)	<0.001

MINS: Myocardial injury after non-cardiac surgery, CCI: Charlson Comorbidity Index, ECMO: Extracorporeal membranous oxygenation

Supplementary Table S4. Baseline characteristics of four quartiles according to the CCI

	**1st quartile (n = 1766)**	**2nd quartile (n = 1662)**	**3rd quartile (n = 1210)**	**4th quartile (n = 995)**	**p-value**
CCI	0.47 (±0.50)	2.00 (±0.00)	3.00 (±0.00)	5.60 (±1.91)	<0.001
Age	65.7 (15.9)	64.8 (13.1)	65.5 (12.5)	65.1 (13.0)	0.248
Male sex	908 (51.4)	1028 (61.9)	753 (62.2)	679 (68.2)	<0.001
Hypertension	1197 (67.8)	1029 (61.9)	803 (66.4)	710 (71.4)	<0.001
Current smoking	219 (12.4)	111 (6.7)	98 (8.1)	78 (7.8)	<0.001
Current alcohol	321 (18.2)	224 (13.5)	153 (12.6)	119 (12.0)	<0.001
History of arrhythmia	174 (9.9)	137 (8.2)	122 (10.1)	136 (13.7)	<0.001
History of heart valve disease	44 (2.5)	30 (1.8)	15 (1.2)	26 (2.6)	0.051
Preoperative care					
Intensive care unit	238 (13.5)	113 (6.8)	116 (9.6)	142 (14.3)	<0.001
ECMO	0 (0.0)	1 (0.1)	0 (0.0)	0 (0.0)	0.496
CRRT	4 (0.2)	20 (1.2)	9 (0.7)	29 (2.9)	<0.001
Ventilator	57 (3.2)	27 (1.6)	18 (1.5)	34 (3.4)	<0.001
Operative variables					
ESC/ESA surgical high risk	404 (22.9)	254 (15.3)	403 (33.3)	364 (36.6)	<0.001
Emergency operation	668 (37.8)	366 (22.0)	269 (22.2)	280 (28.1)	<0.001
General anesthesia	1401 (79.3)	1441 (86.7)	1130 (93.4)	892 (89.6)	<0.001
Operation duration, hours	2.9 (2.4)	3.2 (2.6)	4.1 (2.8)	4.2 (3.2)	<0.001
Continuous infusion of inotropics	718 (40.7)	587 (35.3)	539 (44.5)	474 (47.6)	<0.001
RBC transfusion	137 (7.8)	188 (11.3)	226 (18.7)	294 (29.5)	<0.001
Preoperative use of					
Beta-blocker	514 (29.1)	432 (26.0)	320 (26.4)	411 (41.3)	<0.001
Calcium channel blocker	610 (34.5)	601 (36.2)	419 (34.6)	397 (39.9)	0.027
RAAS inhibitor	697 (39.5)	658 (39.6)	482 (39.8)	433 (43.5)	0.155

Statin	631 (35.7)	546 (32.9)	417 (34.5)	338 (34.0)	0.36
Antiplatelet agent	695 (39.4)	617 (37.1)	469 (38.8)	415 (41.7)	0.13
Direct oral anticoagulant	24 (1.4)	27 (1.6)	25 (2.1)	28 (2.8)	0.041
Warfarin	116 (6.6)	104 (6.3)	60 (5.0)	97 (9.7)	<0.001

Data are presented as *n* (%) or mean (±standard deviation).

CCI: Charlson comorbidity index, ASD: Absolute standardized mean difference, ECMO: Extracorporeal membranous oxygenation, CRRT: Continuous renal replacement therapy, ESC: European Society of cardiology, ESA: European Society of Anaesthesiology, RBC: Red blood cell, RAAS: Renin-angiotensin-aldosterone system

Supplementary Table S5. Clinical outcomes of patients that are not detected as MINS

	**Low CCI group (n = 24205)**	**High CCI group (n = 11981)**	**Unadjusted HR (95% CI)**	**p-value**
30-day mortality	309 (1.3)	144 (1.2)	0.93 (0.76-1.14)	0.480
One-year mortality	1295 (5.4)	1561 (13.0)	2.37 (2.20-2.55)	<0.001
Overall mortality	2732 (11.3)	3389 (28.3)	2.52 (2.40-2.65)	<0.001

MINS: Myocardial injury after non-cardiac surgery, CCI: Charlson Comorbidity Index

† 29394 patients had postoperative troponin measurements that are below the upper reference limit, and 242 patients showed rise of troponin due to non-ischemic etiology.

† Remaining patients without postoperative troponin measurement were not determined.

Supplementary Table S6. Sensitivity analysis of the effect of an unmeasured confounder on hazard ratio of high CCI for 30-day mortality after MINS

		**$OR_{ZY\|X}$**					
		**1.5**	**2**	**2.5**	**3**	**3.5**	**4**
	0.3	1.84 (1.43-2.35)	2.01 (1.57-2.57)	2.15 (1.68-2.75)	2.26 (1.77-2.90)	2.37 (1.86-3.03)	2.45 (1.92-3.13)
	0.4	1.75 (1.37-2.24)	1.87 (1.46-2.38)	1.97 (1.54-2.51)	2.04 (1.60-2.61)	2.11 (1.66-2.69)	2.16 (1.70-2.75)
**OR_{zx}**	0.5	1.70 (1.34-2.16)	1.78 (1.40-2.26)	1.85 (1.45-2.35)	1.91 (1.50-2.43)	1.96 (1.56-2.49)	1.99 (1.56-2.53)
	0.6	1.65 (1.30-2.10)	1.71 (1.35-2.18)	1.77 (1.39-2.25)	1.80 (1.42-2.28)	1.83 (1.44-2.33)	1.86 (1.47-2.37)
	0.7	1.62 (1.28-2.06)	1.66 (1.31-2.11)	1.69 (1.34-2.15)	1.72 (1.35-2.18)	1.75 (1.38-2.22)	1.77 (1.39-2.24)

Prevalence of unmeasured confounder = 40%
Numbers represent HRs (including 95% CIs)

CCI: Charson Comorbidity Index, MINS: Myocardial injury after non-cardiac surgery, OR: Odds ratio, HR: Hazard ratio

X: dichotomous exposure measure, y dichotomous outcome measure, z: potential dichotomous confounder
ORZX indicates the association (OR) between the unmeasured confounder and high CCI
ORZY|X indicates the association (OR) between the unmeasured confounder and 30-day mortality conditional on exposure status

Supplementary Table S7. Weighted score of the CCI

Comorbidities	Assigned weights
Chronic pulmonary disease	1
Diabetes with complications	1
Rheumatologic disease	1
Renal disease	1
Congestive heart failure	2
Dementia	2
Mild liver disease	2
Hemiplegia or paraplegia	2
Any malignancy (leukemia, lymphoma)	2
Moderate to severe liver disease	4
AIDS	4
Metastatic solid tumor	6

CCI: Charlson Comorbidity Index, AIDS: Acquired immune deficiency syndrome
